# Enhanced decontamination of levofloxacin as toxic pharmaceutical residuals from water using CaO/MgO nanorods as a promising adsorbent

**DOI:** 10.1038/s41598-020-71951-6

**Published:** 2020-09-09

**Authors:** Mostafa R. AbuKhadra, Mohamed Gameel Basyouny, Ali A. AlHammadi, Ahmed M. El-Sherbeeny, Mohamed Abdel Salam

**Affiliations:** 1 Geology Department, Faculty of Science, Beni-Suef University, Beni-Suef, Egypt; 2 Materials Technologies and Their Applications Lab, Geology Department, Faculty of Science, Beni-Suef University, Beni-Suef, Egypt; 3 Physics Department, Faculty of Science, Beni-Suef University, Beni-Suef, Egypt; 4 Center for Catalysis and Separations, Khalifa University, P.O.Box 127788, Abu Dhabi, UAE; 5 Chemical Engineering Department, Khalifa University of Science and Technology, P.O. Box 127788, Abu Dhabi, UAE; 6 Industrial Engineering Department, College of Engineering, King Saud University, P.O. Box 800, Riyadh, 11421 Saudi Arabia; 7 Chemistry Department, Faculty of Science, King Abdulaziz University, Jeddah, P.O Box 80200, Jeddah, 21589 Saudi Arabia

**Keywords:** Photocatalysis, Pollution remediation, Nanoparticles

## Abstract

Novel MgO/CaO nanocomposite (MgO/CaO NRs) was synthesized by the hydrothermal method using diatomite porous frustules as a substrate under the microwave irradiation. The composite appeared as well crystalline rod-like nanoparticles with 52.3 nm as average particle size and 112.8 m^2^/g as BET surface area. The synthetic MgO/CaO NRs were addressed as a novel adsorbent for promising removal of levofloxacin (LVX) as pharmaceutical residuals. The adsorption studies revealed effective uptake of levofloxacin by MgO/CaO NRs with theoretical q_max_ of 106.7 mg/g and the equilibrium time of 720 min considering the best pH value (pH 7). The equilibrium studies highly fitted with the Langmuir model of monolayer adsorption considering the values of Chi-squared (χ^2^) and determination coefficient. The estimated adsorption energy from Dubinin–Radushkevich (0.2 kJ/mol) signifies physisorption mechanisms that might be coulombic attractive forces considering the kinetic studies. The thermodynamic addressing for the reactions verified their spontaneous and exothermic nature within a temperature range from 303 to 333 K. Additionally, the prepared MgO/CaO NRs show significant recyclability properties to be used in realistic remediation process and its uptake capacity is higher than several studied adsorbents in literature.

## Introduction

The wide detection of the biologically active chemical compounds in the water resources as contaminants and their metabolites represent one of the main concerns of the contemporary world^1^. The antibiotics are very active biological materials that used widely in human medicine as well as veterinary practice and their existence in water even as traces are of vital impact on the ecosystem and human health^1,2^. Among the commonly studied antibiotic, levofloxacin which is a known type of fluoroquinolone antibiotic was identified extensively in groundwater and surface water as hazardous contaminants^3^. It is used extensively as an antibacterial agent or dysentery, pneumonia, and for the treatment of immunodeficiency virus^2,4,5^. Fluoroquinolone displays very poor metabolic activity in the human body so it commonly released into the water resources and environment as parent compound^5^. The releasing of such toxic, antibacterial, and non-biodegradable properties is of serious negative impacts on human health and the ecosystem. The complexation between the antibiotic as chemical compounds and the other organic or inorganic compounds commonly resulted in the formation of hazardous materials. They are classified as inhibitor chemical compounds and are of negative impacts on the lifestyle of microorganisms and their resistance to bacteria and pathogens^5^.

Thus, the development of advanced materials of promising adsorption and photocatalytic properties to remove levofloxacin from water is of great interest to the environmental and the scientific communities^5^. Numerous materials were applied for this target including MoS_2_ nanosheet decorated by Ag_2_Mo_2_O_7_^5^, goethite^6^, Bi_3_O_4_Cl/BiOCl composite^7^, BiVO_4_-CeVO_4_^4^, biochar^2^, MnO@MnOx^8^, cerium-doped ZnO^9^, graphene oxide-CdS^10^, Ag/AgCl@ZIF-8 modified g-C_3_N_4_^11^, and MoS_2_/TiO_2_^12^. Unfortunately, most of the introduced materials suffer from some disadvantages that may include the high preparation cost, low removal capacities, and complex preparation techniques.

Calcium oxide (CaO) was applied extensively as one of the promising adsorbents in the removal of both organic and inorganic pollutants^13^. It is semiconductor oxide of low cost, environmental properties, high availability, non-toxicity, and high mechanical stability which makes it of promising technical and economic value^14^. It was reported that doping of CaO by metal ions or integrating it with other metal-bearing materials in composites as NiO–CaO, Ag_3_PO_4_–CaO, and CaO–TiO_2_ are of positive influence in enhancing its optical and adsorption properties^15^. Also, magnesium oxide (MgO) was studied widely as one of the best applied adsorptive materials in the removal of several species of toxic contaminants^16^. It was reported that the incorporation of magnesium oxide in hybrid composite with other oxides can improve the chemical reactivity of the product^17^. Thus, the integration between CaO and MgO in a hybrid composite can result in a superior product of effective adsorption activity.

The synthesis of nanostructures based on well-developed porous materials as a template or substrates was investigated in literature as a simple technique to obtained well-developed nanomaterials of high yield, uniform size, and uniform shapes^18^. The diatomite frustules were studied widely as substrates in the synthesis of different types of metal oxides in the nanoscale either as single phases or as composites with the porous structure of diatoms^14,19^. As a geological term, the diatomite term used to refer to a siliceous sedimentary rock composed of diatoms frustules in addition to some natural impurities^14^. The synthetic nanoparticles appeared in different morphologies including nanosheets, nanowires, nanorods, nanospheres, nanotubes, and nanoflakes^20^. The synthetic nanostructures of one-dimensional morphology as the nanorods and nanotubes displayed stunning surface area, high stability, and high dispersion properties in the solid/liquid interface which qualify such materials for effective adsorption applications^19^. Therefore, the introduced study involved the synthesis novel MgO/CaO nanorod structures using diatoms skeleton as a growing substrate under the microwave radiation. The composite was investigated as potential adsorbent for enhanced removal of levofloxacin antibiotic from aqueous solution. The optimization tests in addition to the adsorption mechanisms were discussed in the manuscript.

## Results and discussion

### Characterization

#### XRD investigation and structural properties

**Figure 1:**
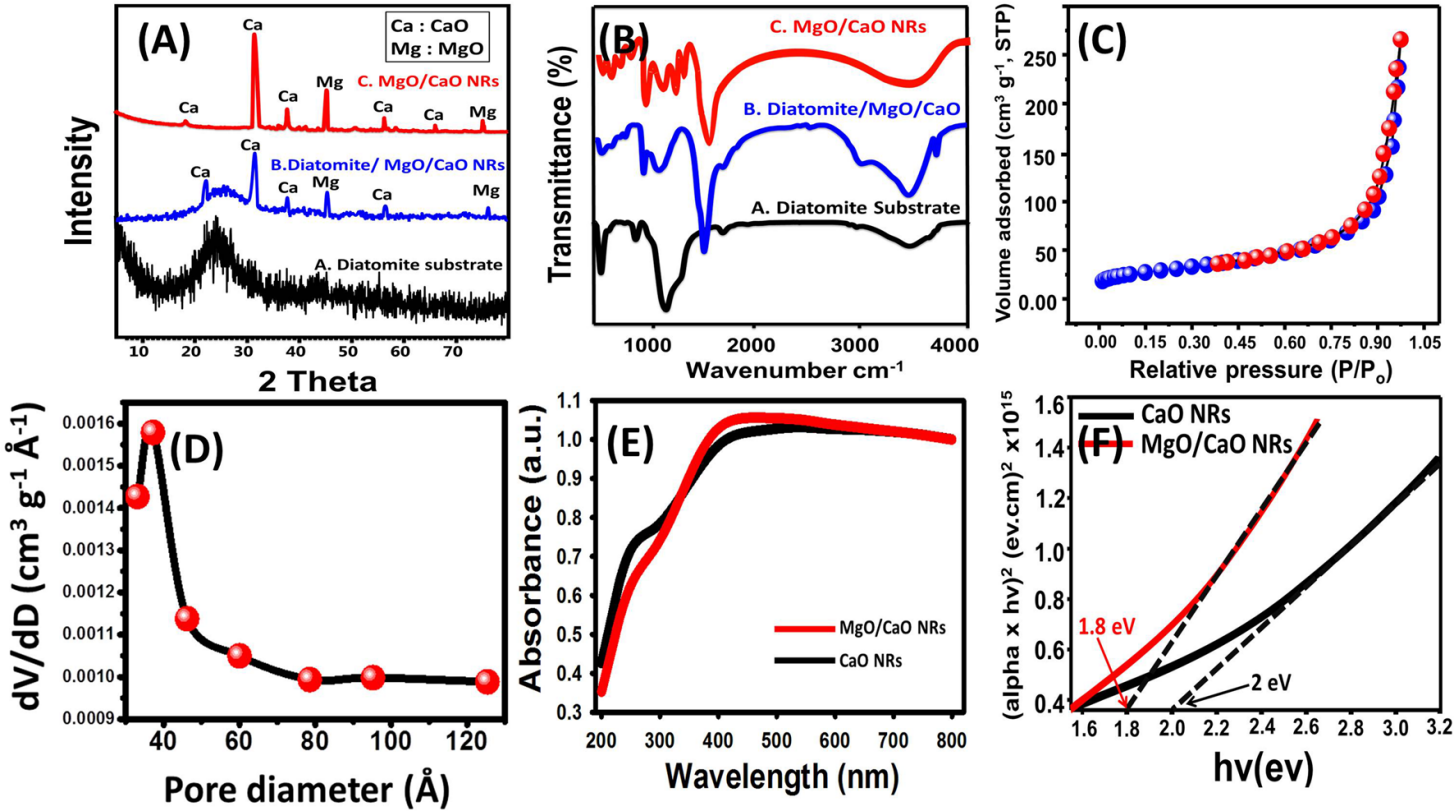
The XRD patterns of diatomite substrate and the synthetic MgO/CaO NRs (**A**), the FT-IR spectra of diatomite substrate, diatomite loaded by MgO/CaO and the synthetic MgO/CaO NRs (**B**), the nitrogen adsorption/desorption isotherm curve of MgO/CaO NRs (**C**), the pore size distribution of MgO/CaO NRs (**D**), the UV–Vis spectra of CaO NRs and MgO/CaO NRs (**E**), and the estimated band gap energies of CaO NRs and MgO/CaO NRs (**F**)^17^.

Diatomite skeletons formed mainly of opaline silica so its XRD pattern showed the distinguished broad peak of amorphous silica around 22° (Fig. 1A.A). The pattern of diatomite/MgO/CaO showed the broad peak of amorphous silica in addition to the characteristic peaks of CaO and MgO (Fig. 1A.B). The pattern of MgO/CaO NRs without the diatomite substrate revealed the dominance of calcium oxide and magnesium oxide as separated phases (Fig. 1A.C). The CaO peaks were observed at 32.24°, 37.6°, 55.41° and 67.14° which corresponding peaks to (111), (200), (202) and (222) planes that characterize the cubic crystal system of CaO (JCPDS card No. 00-004-0777). Also, the principal peaks of MgO were recognized at 43.22° and 74.7° which are the characteristic peaks of (200) and (311) planes that related to the cubic MgO (JCPDS; card No. 01-077-2364) (Fig. 1A.B). The calculated crystallite sizes of MgO and CaO according to Scherrer equation are 16.4 nm and 22.3 nm, respectively.

#### Chemical properties

The FTIR spectrum of diatomite substrate displayed the main bands of amorphous opaline silica (SiO–H (3437 cm^−1^), H–O–H (1638 cm^−1^), symmetrical Si–O–Si (799 cm^−1^), and asymmetrical Si–O-Si (1,092 cm^−1^ and 465 cm^−1^)^21^ (Fig. 1B.A). After loading it by MgO/CaO NRs, the main bands of opaline silica in addition to other bands related to both CaO (Ca–O (545 cm^−1^)) and MgO (Mg–O–Mg (867 cm^−1^)) were detected^14^ (Fig. 1B.B). After the dissolution of the diatomite substrate, the resulted spectrum revealed the disappearance of Si–OH and Si–O–Si that characterize the skeleton of diatoms and all the observed bands are of MgO and CaO structures (Fig. 1B.C). The Ca–O group was identified at 537 cm^−1^ and 718 cm^−1^^22^ (Fig. 1B.C). For MgO, the Mg–O–Mg was recognized at 874 cm^−1^ while the higher and lower frequency stretching of MgO was identified by the bands at 600 cm^−1^ and 420 cm^−1^, respectively^23^ (Fig. 1B.C). The EDX result is of strong agreement with the FT-IR results (40% Ca, 14.7% Mg, and 45.3% O).

#### Textural properties

The nitrogen adsorption/desorption isotherm of MgO/CaO NRs was identified as type-III isotherm that is of the H3 hysteresis loop (Fig. 1C). This type related to plate-like particles of a nanoporous structure formed by the aggregation of the particles^24^ (Fig. 1C). The distribution curve of the present pores reflected the formation of the pores within the size range from 2 nm to about 13 nm and of 4.3 nm as average pore diameter which categorizes MgO/CaO NRs as materials of mesoporous structure (Fig. 1D). Additionally, the MgO/CaO NRs are of 0.103 cm^3^/g average pore-volume, 52.3 nm average particle size, and 112.8 m^2^/g surface area.

#### Optical properties

The optical properties and the bandgap energy of the synthetic MgO/CaO NRs are of vital role in the performance of it for further evaluation of the composite as photocatalyst in the degradation of organic pollutants. The pure phase of CaO NRs and the studied MgO/CaO NRs showed a broad absorption band extended from the UV area to the visible light area (Fig. 1E). This associated with an observable increase in the intensity of MgO/CaO NRs band and slight shifting of it towards the visible light region reflecting enhancing the photocatalytic properties by increasing the produced electron–hole pairs after the integration of CaO by MgO^25,26^ (Fig. 1E). The bandgap energies of both CaO NRs and MgO/CaO NRs were theoretically calculated from Tauc’s equation and the value is 2.1 eV and 1.8 eV, respectively which promising values for future investigation of the composite as photocatalyst (Fig. 1F).

#### Morphological studies

**Figure 2:**
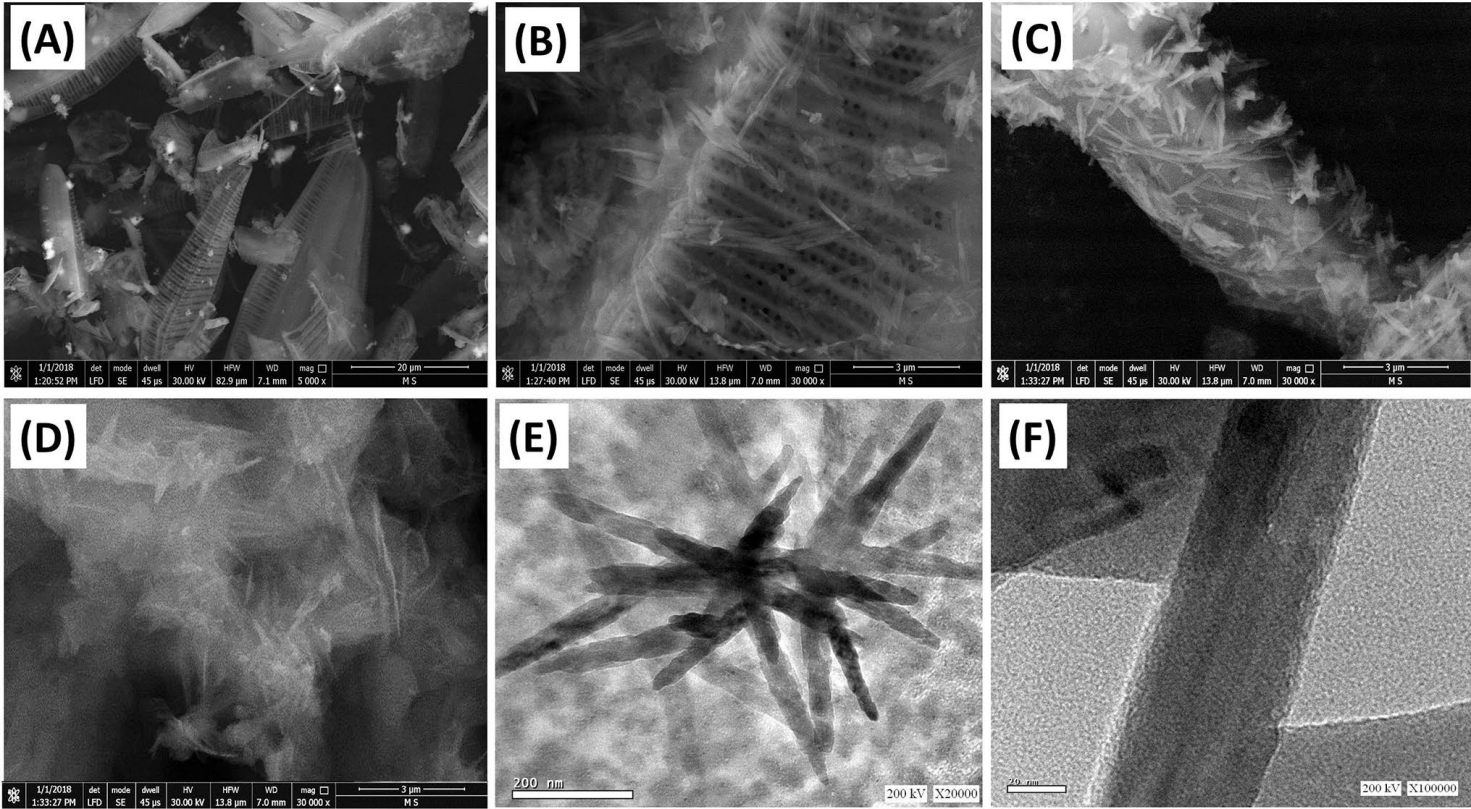
SEM image of diatomite substrate (**A**), SEM images of diatomite substrate with grown MgO/CaO nano-rods (**B**,**C**), SEM images of MgO/CaO nanorods after removing the substrate (**D**), and HRTEM images of the synthetic MgO/CaO nanorods (**E**,**F**)^17^.

The SEM image of refined diatomite substrate showed the presence of pinnate diatomite frustules with their stunning oriented porous structure (Fig. 2A). After the incorporation of the diatomite frustules as the growing substrate, the SEM image revealed decoration of diatomite porous frustules by MgO/CaO nanorods that appeared on random orientation (Fig. 2B,C). After the dissolution of the siliceous diatomite substrate, the produced MgO/CaO NRs appeared as agglomerated fibrous or needle particles aggregated with each other (Fig. 2D). The HRTEM images confirmed the synthesis of MgO/CaO composite as well-developed needle particles or nanorods (Fig. 2E,F). The composite appear as single grains which reflects the homogeneity in the integration process between the two phases. The deep inspection for the main rods reflected their formation as bundles for minor nanorods. This might be related to the effect of the ultrasonic waves during the mixing processes in making the growth of the particles interlocked with each other. The MgO/CaO rods ranged in length from about 200 nm to about 450 nm and ranged in diameter from about 20 nm to about 50 nm.

### Adsorption results

#### Effect of the parameters and optimization

##### Effect of pH

**Figure 3:**
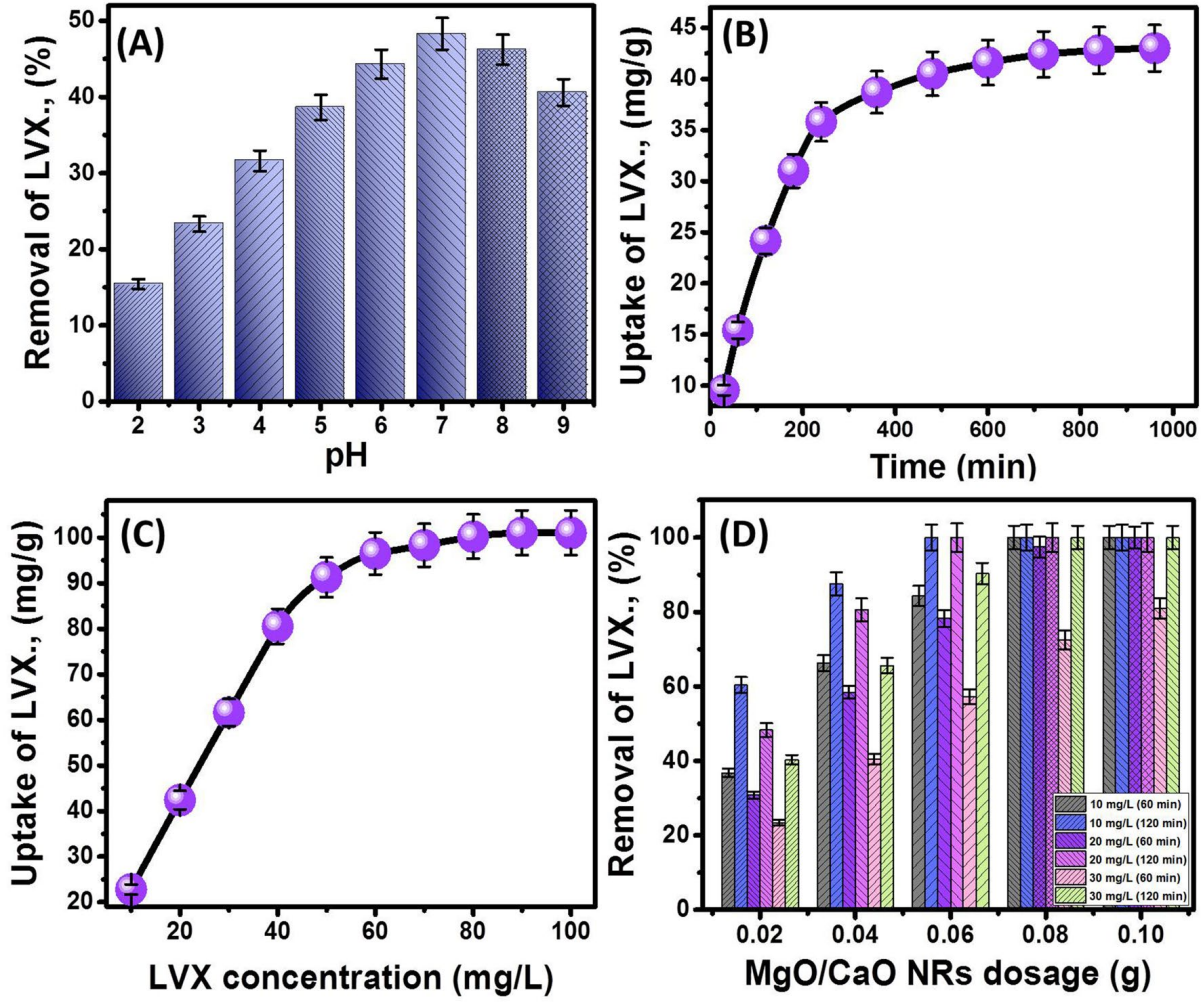
The effect of the adsorption parameters on the uptake of LVX by MgO/CaO NRs, (**A**) the solution pH, (**B**) the time intervals, (**C**) the Levofloxacin initial concentration, and (D) the MgO/CaO NRs dosage.

The adsorption of LVX molecules by MgO/CaO NRs at different pH values showed a significant increase from 14.4 to 48.3% with increasing the pH of the solution from pH 2 to pH 7, respectively (Fig. 3A). The further increase in the LVX solution pH beyond pH 7 resulted in a reduction in the achieved removal percentage to 46.2% and 40.6% at pH 8 and pH 9, respectively (Fig. 3A). Therefore pH 7 was detected as the best value in the MgO/CaO NRs adsorption system for Levofloxacin which is reported also in other studies as Liu et al.^27^, Limbikai et al.^28^, and Yu et al.^29^.

The reported behavior can be explained based on the speciation of Levofloxacin at different pH values as well as the surface properties of the synthetic MgO/CaO NRs. The speciation diagram of LVX revealed the detection of it in three forms (cationic, zwitterionic, and anionic)^27^. The cationic form (protonated piperazinyl group) was detected at pH values lower than pH 5, the anionic form (deprotonated carboxyl group) was identified as the dominant species at pH values higher than pH 8.5, and the zwitterionic form was detected as the dominant species within pH range from pH 5 to pH 8.5^2,6^. Thus the increase in pH associated with the continuous increase in the dominance of the zwitterionic form until achieving its maximum concentration at pH 7 (Yu et al.^29^). At the same time, the protonation of MgO/CaO NRs at the acidic condition and deprotonation of it at the alkaline conditions make its surface positively charged at lower pH values and negatively charged at higher pH values^19,30^. Therefore conducting the reactions at pH lower than or higher than pH 7 causes electrostatic repulsion with the LVX cationic form and anionic form, respectively.

#### Effect of time intervals

As most of the evaluated adsorption system, the uptake of LVX molecules by MgO/CaO NRs with time reflected two stages of different adsorption rates (Fig. 3B). The first stage is of significant changes in the LVX uptake rate and this was reported within a time interval from 30 to 360 min (Fig. 3B). The second LVX uptake stage is of very slight changes in the adsorption rate until attending the equilibrium point of no changes in the amount of adsorbed LVX molecules by MgO/CaO NRs. This was reported within the time intervals from 360 to 960 min achieving the equilibration after 720 min with 42.4 mg/g as experimental equilibrium uptake capacity (Fig. 3B). The observed stages reflected the continuous uptake of LVX molecules by the exposed sites of MgO/CaO NRs until the complete saturation of such sites^31^.

#### Effect LVX concentration

The uptake equilibrium of LVX molecules by MgO/CaO NRs is of vital effect in detecting the maximum adsorption capacity as well as the equilibrium behavior. The adsorption of LVX as a function of its initial concentrations displayed an obvious increase in the value was testing higher concentrations of it until achieving its maximum adsorption capacity without possible enhancement for the obtained value even with the further increase in the LVX concentration (Fig. 3C). The maximum LVX adsorption capacity (100 mg/g) was recognized after treating 80 mg/L of LVX which can be signified as the equilibrium concentration at which all the receptor sites of MgO/CaO NRs were occupied by the LVX molecules (Fig. 3C). The LVX adsorption equilibrium curve is of L-type (2L) type which identified for adsorbents that exhibit a very high affinity towards the studied pollutants^32,33^.

#### Effect of the used MgO/CaO NRs quantities

The influence of the used MgO/CaO NRs was studied for different concentrations (10 mg/L to 30 mg/L) and two selected time intervals (60 min and 120 min) (Fig. 3D). The results demonstrated an enhancement in the recognized removal percentages of LVX for all the studied concentrations and the selected time intervals with the controlled increase in the MgO/CaO mass from 0.02 to 0.1 g (Fig. 3D). This is a commonly reported phenomenon in the adsorption systems and related to the associated enrichment in the available receptor sites of MgO/CaO NRs as well as its exposed surface area with mixing higher masses of it with the solution^19,31^. The reported results reflected complete removal of 10 mg/L of LVX after 60 min and 120 min by using 0.08 and 0.06 g of MgO/CaO NRs, respectively (Fig. 3D). Also, 20 mg/L of LVX antibiotic can be completely removed after 60 min and 120 min by increasing the mass to 0.1 g and 0.06 g, respectively (Fig. 3D). Such effective results were recognized also for 30 mg/L of LVX and the complete removal was achieved after 120 min using 0.08 g of MgO/CaO NRs (Fig. 3D). Such removal percentages give the composite promising importance to remove high concentrations of LVX within short periods.

#### Kinetic, equilibrium, and thermodynamic studies

##### Kinetic studies

**Figure 4:**
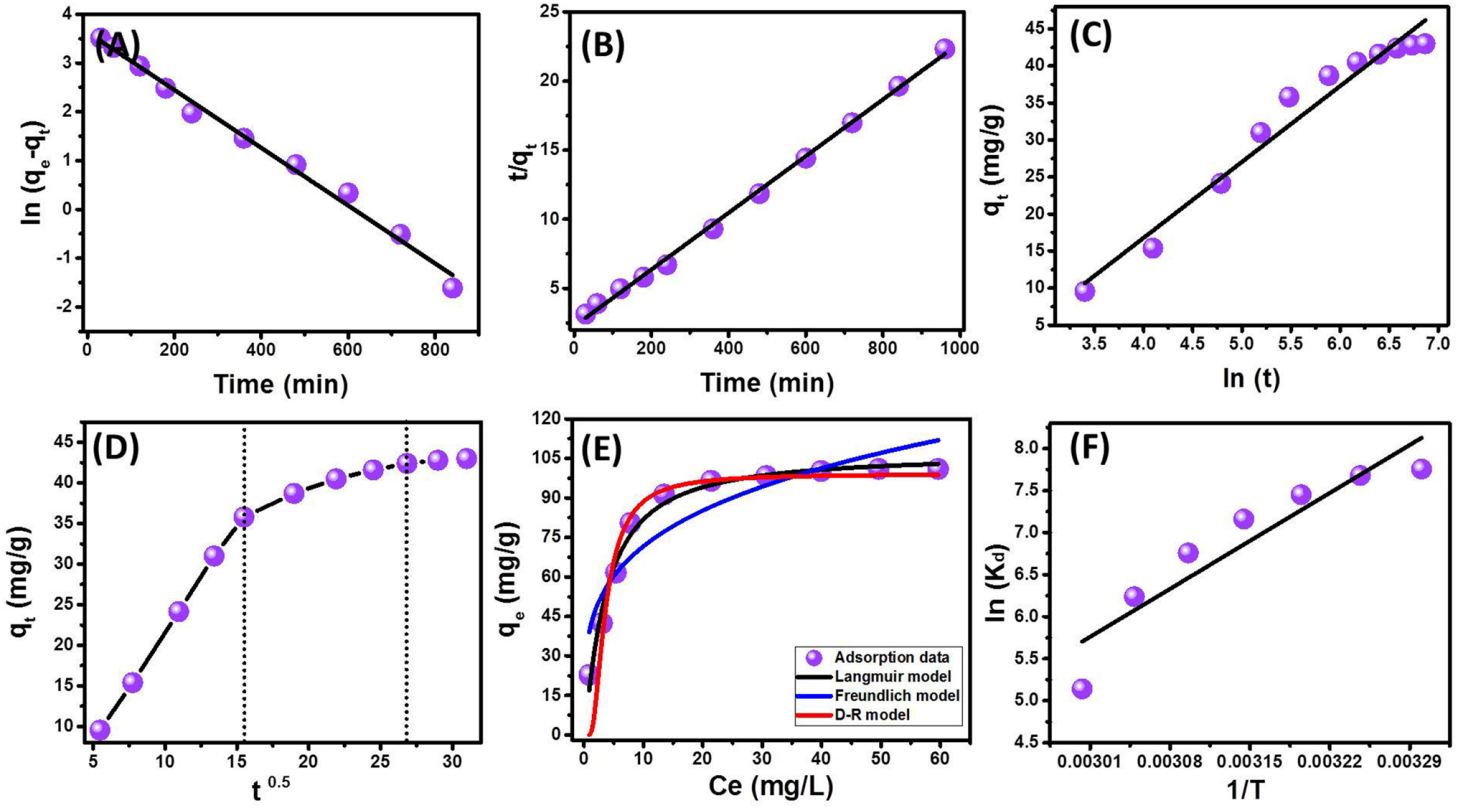
Fitting the adsorption results of LVX by MgO/CaO NRs with Pseudo-first order kinetic model (**A**), Pseudo-second order kinetic model (**B**), Elovich kinetic model (**C**), Intraparticle diffusion kinetic model (**D**), Langmuir, Freundlich and D-R isotherm models (**E**), and Van’t Hof equation (**F**).

The kinetic behavior of LVX molecules in the MgO/CaO NRs adsorption system was addressed considering the assumptions of four theoretical (Pseudo-First order, Pseudo-Second order, Elovich, and Intraparticle diffusion models) (Fig. 4A–D). The representative equations of the inspected models were listed in Table S1. The fitting results reflected strong agreement for the kinetic behavior of LVX in MgO/CaO NRs adsorption system with the theoretical assumption of Pseudo-first and Pseudo-second order models as well as the Elovich model (Fig. 4A–C; Table 1). The pseudo-first-order model related normally to physical adsorption processes while the Pseudo-second order model related to several mechanisms of more chemical nature including electron sharing, surface complexation, electron exchange, and internal diffusion^19^. The Elovich model also suggested more chemical behavior for the adsorption of LVX within energetically heterogeneous materials which can be detected for composites materials as MgO/CaO NRs^19,34^. The fitting results suggested complex uptake mechanisms of LVX by MgO/CaO NRs that can be supported by the further equilibrium models.

**Table 1:** The estimated theoretical parameters of the kinetic models, isotherm models, and the thermodynamic studies.

Kinetic models			Isotherm models		
Model	Parameters	LVX	Model	Parameters	LVX
Pseudo-first order	k_1_ (mg/min)	0.006	Langmuir	q_max (mg/g)_	106.75
				b(L/mg)	0.219
	qe (Cal) (mg/g)	37.73		R^2^	0.98
	R^2^	0.98		R_L_	0.044–0.313
				***X***^**2**^	0.16
Pseudo-Second order	k_2_ (mg/min)	1.86 × 10^–4^	Freundlich	1/n	0.249
				K_F_	40.31
				R^2^	0.83
	qe (Cal) (mg/g)	48.78		*X*^2^	0.321
	R^2^	0.99			
Elovich	β (g/mg)	0.0976	Dubinin–Radushkevich	β (mol^2^/KJ^2^)	12.35
				q_m_ (mg/g)	99.14
	α (mg/g min)	108.65		R^2^	0.90
				E (KJ/mol)	0.2
	R^2^	0.96			

Parameters	Thermodynamic parameters				
	Temperature (K)	LVX			
∆G° (kJ mol^−1^)	303	− 19.54			
	308	− 19.64			
	313	− 19.42			
	318	− 18.95			
	323	− 18.12			
	328	− 17.01			
	333	− 14.24			
ΔH° (kJ mol^−1^)		− 67.78			
ΔS° (J K^−1^ mol^−1^)		− 156.15			

Intra-particle-diffusion model also was addressed to introduce more physical explanations for the kinetic properties of the studied MgO/CaO NRs adsorption system for LVX molecules (Fig. 4D). The detection of the curves as three LVX uptake segments without intersection with the original point revealed the operation of more than one mechanism during the uptake of LAVX by MgO/CaO NRs and did not control only by the diffusion of the dissolved LVX molecules^31^ (Fig. 4D). The first segment related to the external adsorption of LVX by the external receptor sites of MgO/CaO NRs and this stage represents the dominant process. The second segment related to the layered adsorption stage of limited intra-particle diffusion for the target ions. The third segment reflected the equilibrium or the saturation state of MgO/CaO NRs which formed by forming thick layers of the adsorbed LVX molecules on its surface where the dominant mechanisms are inter-ionic attraction and molecular association^35^.

#### Isotherm studies

The equilibrium of MgO/CaO NRs adsorption system for LVX molecules was addressed considering the nonlinear fitting results with the suppositions of Langmuir, Freundlich, and Dubinin–Radushkevich models (Fig. 4E) and their represented equations were listed in Table S1. The Langmuir assumption suggested the adsorption of LVX as dissolved molecules on the surface of MgO/CaO NRs in a monolayer form by receptor sites distributed homogeneously on the surface of the synthetic nanorods^36^. The Freundlich assumption related to heterogeneous uptake of the LVX molecules in multilayer form^37^. The fitting results showed excellent correlation coefficient value with the Langmuir model as compared to the obtained value for the Freundlich model which was supported by the Chi-squared (χ^2^) values (Table 1). Additionally, the theatrical values RL parameter (0.044–0.313) and the theoretical LVX maximum uptake capacity reflected the applicability of MgO/CaO NRs to adsorb about 106.75 mg/g in monolayer from by homogeneous adsorption process^38^ (Table 1).

The Dubinin–Radushkevich (D-R) is an important model for its value in understating the nature of the controlling reaction during the uptake of LVX molecules (physical and chemical) considering based on the Gaussian energy^19^. The LVX adsorption data showed strong agreement with the D-R model and its related parameters displayed 99.14 mg/g as maximum uptake capacity and 0.2 kJ/mol as adsorption energy which very low value and suggested strong physisorption process (Table 1). The kinetic and isotherm studies suggested the adsorption of LVX molecules by MgO/CaO NRs by coulombic attractive forces that involves ion exchange process with very weak electrostatic attraction without formation or destruction of chemical bonds which explain the fitting of the results with Pseudo-second order kinetic model^36^.

#### Thermodynamic studies

The thermodynamic properties of MgO/CaO NRS adsorption system for LVX were studied considering the theoretical values Gibbs free energies (∆G°), enthalpy (ΔH°) and the entropy (ΔS°). The value of ∆G^o^ was obtained from Eq. (1) while the values of ΔH° and ΔS° were estimated as theoretical parameters for the linear fitting with Van’t Hof equation (Eq. (2))^36^ (Fig. 4F).

1 $${\Delta G}^{0}=-RT In {K}_{d}$$

2 $$\mathrm{In }\left({K}_{c}\right)=\frac{{\Delta S}^{o}}{R}-\frac{{\Delta H}^{o}}{RT}$$

The reported negative signs for ∆G^o^ within temperature from 303 to 318 K reflected the uptake of LVX by MgO/CaO NRs through spontaneous reactions of favorable properties and this declined with accomplishing the reactions at temperature beyond 318 K^37^ (Table 1). The negative value of (ΔH°) revealed the nature of the LVX uptake reaction as an exothermic reaction and its randomness properties decrease significantly with increasing the temperature which reflected in the negative sign of ΔS°^19^ (Table 1). The value of free energy and enthalpy supported the physisorption mechanism for the uptake of LVX by MgO/CaO NRs.

### Reusability

**Figure 5:**
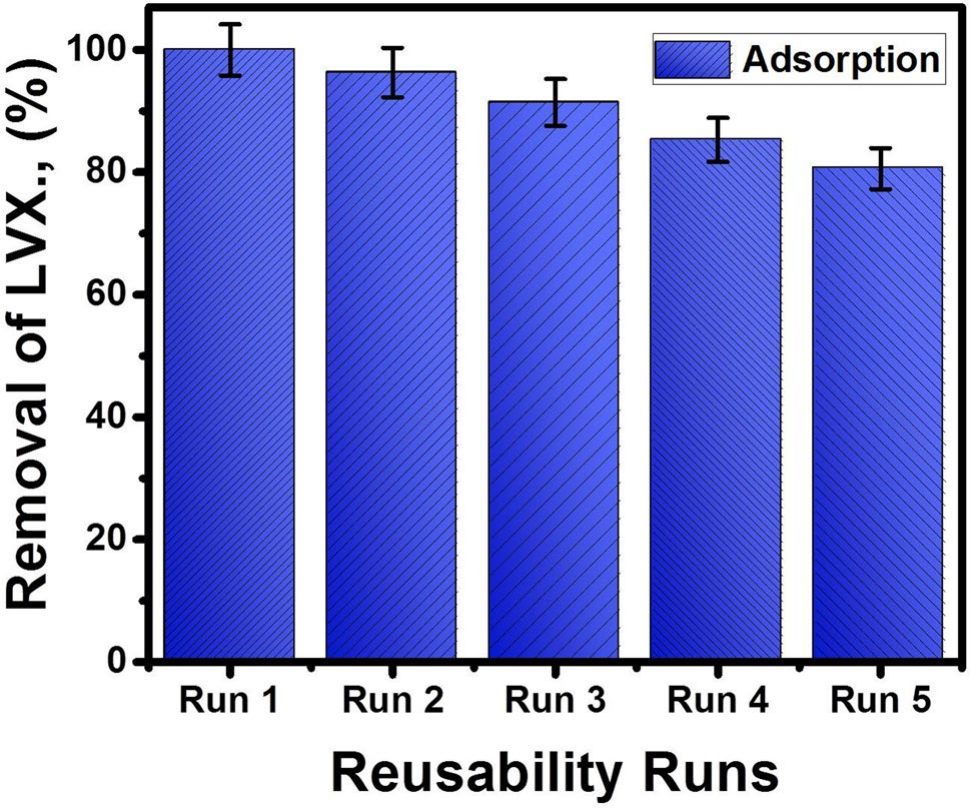
The reusability properties of MgO/CaO NRs to be used for five runs in the adsorption and photocatalytic removal of LV.

The reusability properties of MgO/CaO NRs as adsorbent to be reused in the removal of LVX several times were inspected for five reusing cycles (Fig. 5). The reusing test was performed using 0.1 g of the synthetic nanorods in the removal of 20 mg/L of LVX for 120 min at pH 8 and 30 °C as temperature (Fig. 5). The composite after each reusability test was washed extensively with distilled water five times and dried in an oven at 80 °C for 8 h as the regeneration technique. The composite displayed significant reusability as an adsorbent with removal percentages higher than 80% for the adsorption tests (Fig. 5). The achieved removal percentage by adsorption decreased by 100%, 96.3%, 91.4%, 85.3%, and 80.6% with repeating the reusing runs by run1, run 2, run 3, run 4, and run 5, respectively (Fig. 5). This gives the synthetic MgO/CaO NRs high reusability value which qualifies it for commercial applications.

### Effect of MgO integration

**Figure 6:**
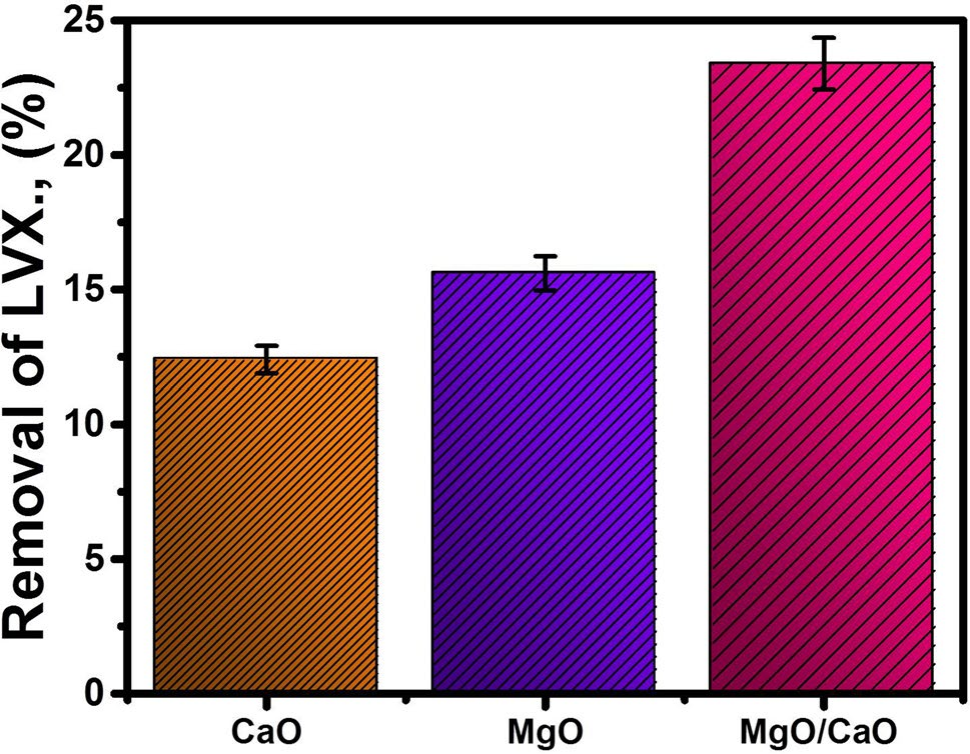
The effect of the integration between MgO and CaO in the adsorption removal of LVX.

The influence of the integration between CaO and MgO as heterogeneous nanostructure in enhancing the adsorption removal of LVX molecules was studied for 30 mg/L of LVX using 0.02 g of MgO/CaO NRs at pH 7 for 120 min (Fig. 6). The study was conducted as a comparison study between CaO and MgO as single phases and the results plotted in Fig. 6. The adsorption results showed removal for the LVX molecules (20 mg/L) by 12.4%, 15.6%, and 23.4% using CaO, MgO, and MgO/CaO NRs, respectively. This demonstrates enhancement in the adsorption properties of MgO/CaO NRs by 11% and 7.8% than the achieved results by CaO and MgO, respectively (Fig. 6).

### Comparison study

The adsorption capacity of the synthetic MgO/CaO NRs for the removal of LVX molecules were compared with other studied adsorbents in the literature (Table 2). The synthetic composite displayed higher adsorption capacity than some natural clay adsorbents (kaolinite and montmorillonite), modified clay (iron pillared montmorillonite), natural, modified, and synthetic zeolite (mordenite, Zeolite-Y, and ZSM-5), carbon-based materials (charcoal and biochar), and some advanced synthetic materials (Fe_3_O_4_@SiO_2_ and MIL-100(Fe)) (Table 2). Such results demonstrate the excellent technical value of MgO/CaO NRs as adsorbent for the LVX molecules as toxic pharmaceutical residuals from water.

**Table 2:** Comparison between the synthetic MgO/CaO NRs and other adsorbents as function of their maximum adsorption capacities.

Adsorption		
Adsorbent	qe (max)	References
Charcoal	87	^39^
Mechanochemistry treated zeolite	47.68	^40^
Kaolin	0.26	^39^
Goethite	1.03	^41^
Zeolite Y	45	^42^
Fe_3_O_4_@SiO_2_	6.848	^43^
Mordenite	27	^42^
ZSM-5	16	^42^
MIL-100(Fe)	87.34	^44^
MgO/CaO NRs	106.75	This study

## Conclusion

MgO/CaO nanorods were synthesized as a novel nanocomposite using the porous diatomite frustules as a substrate. The composite is of high surface area (112.8 m^2^/g) and promising adsorption activity in the removal of levofloxacin from water. It is highly dependent on the pH value, of 106.7 mg/g as maximum adsorption capacity and 720 min as equilibrium time. The adsorption system was represented very well by the Langmuir model suggesting monolayer uptake of levofloxacin and the adsorption energy (0.2 kJ/mol) suggested a physisorption mechanism. The synthetic MgO/CaO NRs is of high stability and achieved better results than several studied adsorbents.

## Experimental work

### Materials

Refined diatomite of chemical composition (98.4% SiO_2_, 1.14% Al_2_O_3_, and 0.46% L.O.I) was used as a synthesis substrate and was delivered by the Central Metallurgical and Development, Egypt. Analytical grade NaOH pellets, magnesium nitrate hexahydrate (Mg (NO_3_)_2_·6H_2_O), and calcium nitrate tetrahydrate (Ca (NO_3_)_2_·4H_2_O) (> 98%, Sigma Aldrich) were used as the main chemical precursors in the fabrication of the MgO/CaO nanorods. Levofloxacin antibiotic (C_18_H_20_N_3_O_4_F) (> 98.0%, Sigma Aldrich) was used to prepare the polluted solution for the adsorption tests.

### Synthesis of MgO/CaO nanorods (MgO/CaO NRs)

The synthesis process involved homogenous dispersion of diatomite fractions (5 g) in deionized water (50 mL) under a complex mixing system including magnetic stirring (500 rpm) as well as ultrasonic irradiation (240 W (60% power)). Another mixture of Ca(NO_3_)_2_·4H_2_O (0.5 M) and Mg(NO_3_)_2_·6H_2_O (0.5 M) was prepared under constant stirring to preserve the homogeneity between the reacting components. After that, the NaOH solution (1 M) was added slowly to Mg/Ca mixture under constant stirring at a fixed temperature of 65 °C until the formation of a dense white gel. Then, the formed gel was mixed homogenously with the pre-dispersed diatomite fractions under a complex mixing system (500 rpm stirring 60% ultrasonic power) for 2 h to confirm the formation of the homogenous mixture. Then, the finally produced mixture was transferred into cylindrical Teflon autoclave under domestic microwave irradiation (900 W) for 30 min to form diatomite skeletons supported by MgO/CaO nanorods. The used diatomite substrate was dissolved by the alkaline dissolution process at pH 10 for 2 h and then the pH was neutralized by washing the material five times using deionized water. Finally, the product was heated at 850 °C for 2 h to confirm the existence of the formed crystalline phases as oxides.

### Characterization

The refined diatomite substrates, as well as the fabricated MgO/CaO nanorods, were addressed considering their X-ray diffraction patterns which were acquired via X-ray diffractometer (PANalytical-Empyrean type). The morphological examination was accomplished using Scanning-Electron Microscope (Gemini, Zeiss-Ultra 55) and Transmission Electron Microscope (JEOL-JEM, 2100). The chemical properties of the MgO/CaO nanorods were identified by the energy dispersive X-ray system (EDX) also the main chemical groups were recognized by the FT-IR Raman spectrometer (Vertex-70 type). The textural parameters of pore size distribution, pore-volume, and the surface were estimated by Brunauer–Emmett–Teller (BET) and Barrett–Joyner–Halenda (BJH) methods. The optical properties of MgO/CaO nanorods were studied based on the light absorption spectrum within the area from 200 to 900 nm which was obtained by the UV–Vis spectrophotometer (Shimadzu-M160 PC).

### Removal of LXX

#### Adsorption system

The adsorption capacity of MgO/CaO NRs for the Levofloxacin molecules (LVX) was studied considering the main controlling factors as the pH, time interval, LVX concentration, MgO/CaO NRs mass, and the temperature. The best adsorption pH for LVX by MgO/CaO was studied after a series of tests at different pH values from pH 2 to pH 9. During the experiments, the main conditions were fixed at 120 min time, 0.02 g of MgO/CaO as used adsorbent quantity, 20 mg/L as LVX concentration in 50 mL distilled water, and 30 °C as temperature. The kinetic behavior was followed by an investigation of the uptake of LVX at different periods (30 min to 960 min) using 0.02 g of MgO/CaO NRs as used adsorbent quantity and 20 mg/L of LVX at pH 7 and 30 °C as temperature. Also, the equilibrium studies were accomplished by studying the uptake of LVX molecules at different concentrations of it (10 mg/L to 100 mg/L) using 0.02 g of MgO/CaO NRs as used adsorbent quantity and at pH 7 and 30 °C as temperature for 960 min. Additionally, the thermodynamic properties MgO/CaO NRs adsorption system for LVX was evaluated within a temperature range from 303 to 333 K considering the concentration at 20 mg/L, the volume at 50 mL, the pH at pH 7 and the time at 120 min. Finally, the influence of MgO/CaO NRs quantities in inducing the removal percentages of LVX was studied using different quantities of it (0.02 g to 0.1 g) for different time intervals and different LVX concentrations (10 mg/L to 30 mg/L) for two selected time intervals (60 in and 120 min) at pH 7 and 30 °C as temperature.

### The analytical techniques of LVX

The removal results either by adsorption tests were calculated considering the obtained results from the analysis of the treated samples by HPLC system (Merck/Hitachi). The conducted adsorption tests were triplicated and the discussed results are the average values of standard deviation less than 4.54%.

## Supplementary information

Supplementary Information

## Abbreviations

NRs: Nanorods
LVX: Levofloxacin
t: Time (min)
∆G^o^: Gibbs free energy (kJ mol^−^^1^)
ΔH°: Standard enthalpy (kJ mol^−^^1^)
ΔS°: Entropy (kJ mol^−^^1^)
R: Gas constant
T: The absolute temperature
K_c_: Langmuir constant
