# Supplementary material for: Enhanced decontamination of levofloxacin as toxic pharmaceutical residuals from water using CaO/MgO nanorods as a promising adsorbent
Source: Sci Rep. 2020 Sep 9;10:14828. doi: 10.1038/s41598-020-71951-6 (PMC7481205; doi:10.1038/s41598-020-71951-6)
Supplement: Supplementary file 1 — Supplementary Information [file 41598_2020_71951_MOESM1_ESM.docx]

**Enhanced decontamination of levofloxacin as toxic pharmaceutical residuals from water using CaO/MgO nanorods as promising adsorbent**

**Mostafa R. AbuKhadra٭ ^1, 2^, Mohamed Gameel Basyouny ^2,3^, Ali A. AlHammadi ^4,5^, Ahmed M. El-Sherbeeny^6^, Mohamed Abdel Salam^7^**

^1^Geology Department, Faculty of Science, Beni-Suef University, Beni-Suef city, Egypt

^2^Materials Technologies and their Applications Lab, Geology Department, Faculty of Science, Beni-Suef University, Beni-Suef City, Egypt

^3^Physics Department, Faculty of Science, Beni-Suef University, Beni-Suef city, Egypt

^4^Center for Catalysis and Separations, Khalifa University, P.O.Box 127788, Abu Dhabi, UAE

^5^Chemical Engineering Department, Khalifa University of Science and Technology, P.O. Box 127788, Abu Dhabi, United Arab Emirates

^6^Industrial Engineering Department, College of Engineering, King Saud University, P.O. Box 800, Riyadh 11421, Saudi Arabia

^7^Chemistry Department, Faculty of Science, King Abdulaziz University, Jeddah, P.O Box 80200-Jeddah 21589, Kingdom of Saudi Arabia

Corresponding author Email: Abukhadra89@Science.bsu.edu.eg (M.R. Abukhadra*),

**Table.S1.** the representative equations of the studied kinetic and isotherm model and their parameters

| Kinetic models | | |
| --- | --- | --- |
| Model | **Linear equation** | **Parameters** |
| Pseudo-first-order | $\ln(q_{e}-q_{t})=ln q_{e}-k_{1}t$ | q_t_ (mg/g) is the adsorbed LVX at time (t), and K_1_ is the rate constant of the first-order adsorption (min^-1^) |
| Pseudo-second-order | $\frac{t}{q_{t}}=\frac{1}{K_{2}q_{e}^{2}}+\frac{t}{q_{e}}$ | qe is the quantity of adsorbed LVX after equilibration (mg/g), and K_2_ is Lagergren model rate constant (g/mg min). |
| Elovich model | $q_{t}=\frac{1}{\beta}\ln(\alpha\beta)+\frac{1}{\beta} In \left( t \right)$ | α is the initial adsorption rate (mg/min) and β is the surface saturation (g/mg) |
| Intra-particle diffusion | $q_{t} =K_{P}t^{1/2} + C$ | k_p_ (mg g^-1^ min ^-0.5^) is the intraparticle diffusion rate constant and C is the intercept of the line |
| Isotherm models | | |
| Model | **Equation** | **Parameters** |
| Langmuir | $\frac{C_{e}}{q_{e}} =\frac{1}{\mathrm{bq}_{\max}}+\frac{C_{e}}{q_{\max}} (Linear)$  $q_{e}=\frac{q_{max} bC_{e}}{(1+bC_{e})} (Nonlinar)$ | *C_e_* is the rest LVX concentrations (mg/L), *q_max_* is the theoritical maximum adsorption capacity (mg/g), and *b* is the Langmuir constant (L/mg) |
| Freundlich | $Log qe = (1/n) log Ce+log K_{f}$ (*Linear*)  $q_{e}=K_{f}C_{e}^{1/n}$ *(Nonlinear)* | K_F_ is the constant of Freundlich model related to the adsorption capacity and n is the constant of Freundlich model related to the adsorption intensities |
| Dubinin–Radushkevich | $\ln(qe) =\ln(q_{m})-\betaɛ^{2} (Linear)$  $q_{e}=q_{m}e^{-\betaɛ^{2}}$ *(Nonlinear)* | β (mol^2^/KJ^2^) is the D-R constant, ɛ (KJ^2^/mol^2^) is the polanyil potential, and q_m_ is the adsorption capacity |
